# Patient safety indicators for England from hospital administrative data: case-control analysis and comparison with US data

**DOI:** 10.1136/bmj.a1702

**Published:** 2008-10-17

**Authors:** Veena S Raleigh, Jeremy Cooper, Stephen A Bremner, Sarah Scobie

**Affiliations:** 1 Healthcare Commission, London EC1Y 8TG; 2 University of Surrey, Guildford, Surrey GU2 7WG; 3 National Patient Safety Agency, London W1T 5HD

## Abstract

**Objective** To assess the feasibility of deriving patient safety indicators for England from routine hospital data and whether they can indicate adverse outcomes for patients.

**Design** Nine patient safety indicators developed by the United States Agency for Healthcare Research and Quality (AHRQ) were derived using hospital episode statistics for England for 2003-4, 2004-5, and 2005-6. A case-control analysis was undertaken to compare length of stay and mortality between cases (patients experiencing the particular safety event measured by an indicator) and controls matched for age, sex, health resource group (standard groupings of clinically similar treatments that use similar levels of healthcare resource), main specialty, and trust. Comparisons were undertaken with US data.

**Setting** All NHS trusts in England.

**Participants** Inpatients in NHS trusts.

**Results** There was fair consistency in national rates for the nine indicators across three years. For all but one indicator, hospital stays were longer in cases than in matched controls (range 0.2-17.1 days, P<0.001). Mortality in cases was also higher than in controls (5.7-27.1%, P<0.001), except for the obstetric trauma indicators. Excess length of stay and mortality in cases was greatest for postoperative hip fracture and sepsis. England’s rates were lower than US rates for these indicators. Increased length of stay in cases was generally greater in England than in the US. Excess mortality was also higher in England than in the US, except for the obstetric trauma indicators where there were few deaths in both countries. Differences between England and the US in excess length of stay and mortality were most marked for postoperative hip fracture.

**Conclusions** Hospital administrative data provide a potentially useful low burden, low cost source of information on safety events. Indicators can be derived with English data and show that cases have poorer outcomes than matched controls. These data therefore have potential for monitoring safety events. Further validation, for example, of individual cases, is needed and levels of event recording need to improve. Differences between England and the US might reflect differences in the depth of event coding and in health systems and patterns of healthcare provision.

## Introduction

Safety of patients is an international problem: reviews of case notes have established that 4-16% of patients admitted to hospital experience an adverse event.^1^ ^2^ ^3^ Definitions of safety vary but usually encompass the “avoidance, prevention, and amelioration of adverse outcomes or injury from the process of health care.”^4^

With growing international interest in patient safety, there is increasing need to monitor the safety of organisations and evaluate safety initiatives. Measuring the scale and impact of safety incidents, however, is a major challenge, and estimates of deaths caused by such incidents vary widely.^5^ Relevant studies are costly to undertake, and the findings depend on thresholds used for including events.^6^ ^7^ There has been considerable investment in local and national reporting systems, and, although these are a valuable resource for learning, voluntary reporting systems are unlikely to provide systematic and reliable information for monitoring patient safety because many incidents go unreported.^8^ Routine data sources have potential for identifying patient safety incidents, with the advantage of no additional data collection costs and burden.

We examined the feasibility of deriving patient safety indicators from hospital episode data for England, whether the indicators point to adverse outcomes for patients, and how the results compare with data from the United States. We used a set of patient safety indicators that were designed to screen administrative data for events that indicate a potentially preventable problem of patient safety and were developed by the US Agency for Healthcare Research and Quality (AHRQ).^9^ The original AHRQ indicators have undergone several phases of development and refinement since being launched in 2003. They have been developed and evaluated with input from clinician panels, expert coders, empirical analysis, and feedback from users.^10^ The indicators have been used extensively in the US for national and local quality improvement and safety measurement initiatives.^10^ The development of patient safety indicators in England is running in parallel with international efforts to derive comparative indicators of patient safety.^11^

## Methods

### Selection of indicators

Of the 29 AHRQ patient safety indicators, we selected nine for analysis in this first phase (the denominators, shown in parentheses, have exclusions as per the detailed AHRQ specifications):

- Death in low mortality healthcare resource groups (low mortality healthcare resource groups spells)
- Iatrogenic pneumothorax (discharges)
- Decubitus ulcer (discharges with a length of stay of five or more days)
- Selected infections due to medical care (discharges)
- Postoperative hip fracture (surgical discharges)
- Postoperative sepsis (elective surgery discharges in patients aged 18 or over with a length of stay of over three days)
- Obstetric trauma with third/fourth degree lacerations—vaginal with instrument (instrument assisted vaginal deliveries)
- Obstetric trauma with third/fourth degree lacerations—vaginal without instrument (vaginal deliveries without instrument assistance)
- Obstetric trauma with third/fourth degree lacerations—caesarean delivery (caesarean deliveries) (this could arise when there is a trial of labour with instrumental assistance, which subsequently results in a caesarean delivery).

The choice of the nine indicators was informed by the following considerations: relative feasibility/complexity of coding conversion, potential reliability of coding in hospital episode statistics, and safety priorities for the Healthcare Commission (for example, maternity, infection control). The derivation and analysis of this set of indicators will inform development of the remaining indicators.

### Data used for analysis

We used hospital episode statistics for the financial years 2003-4, 2004-5, and 2005-6 for the analysis. The statistics comprise an administrative dataset of all NHS inpatients in England, covering about 13 million episodes of care annually. They contain demographic, administrative, and clinical (primary/secondary diagnoses, primary/secondary procedures, outcomes) details for every inpatient receiving NHS care. Episodes of consultant care were linked to form hospital spells.

The specifications of the US patient safety indicators use ICD-9 (international classification of diseases, ninth revision): each indicator is defined by specific numerator and denominator codes. The hospital episode statistics, however, are based on ICD-10 codes for diagnoses and Office of Population Censuses and Surveys (OPCS) codes for procedures. We translated the ICD-9 code specifications into ICD-10 and OPCS codes using semi-automated text word searches and manual coding, with the aim of obtaining the “best fit.” Health resource groups are standard groupings of clinically similar treatments that use similar levels of healthcare resource. We used health resource groups v3.5 in the analysis. Details of the coding used in this paper are available on request.

### Statistical analysis

Statistical analysis explored whether hospital episode statistics are suitable for such analyses and whether the resulting indicators were likely to indicate adverse outcomes for patients.

We calculated event rates at national level for each indicator and compared them across the three years with a view to testing the underlying suitability of hospital episode statistics for such analyses. Wide year on year variation in the results would suggest erratic coding of events in the hospital episode statistics and their unsuitability for deriving AHRQ safety indicators; whereas consistency across years (either unchanging or showing a trend) would satisfy the initial screen for potential fitness for purpose of the data.

We also analysed length of stay and mortality in cases (patients experiencing the particular safety event measured by an indicator) and matched controls (where such an event did not occur) for each indicator except death in low mortality health resource groups to establish whether or not the results indicated that an adverse event had occurred among cases. If the cases were patients who had suffered an adverse event, the expectation is that they would have longer hospital stays and higher mortality. If an indicator reflected only arbitrary variations in coding practice or underlying morbidity, we would expect no systematic differences in length of stay or mortality between patients with and matched patients without a recorded event. To take account of the underlying clinical complexity of cases, we undertook a matched case-control analysis. Each case was matched with up to four controls for age (within five years either side of case), sex, health resource groups (a derived measure of use of healthcare resources commonly used to adjust for case mix), main specialty, and trust. A control could be matched to only one case; if more than four controls matched a case, we randomly selected four. We calculated the mean length of stay for controls per case and subtracted it from the length of stay for that case. We then calculated the mean difference in length of stay between cases and controls. We used paired *t* tests to see if this was significantly different from zero. Similarly, the difference in percent mortality between cases and controls was derived and tested for significance. (The indicator on death in low mortality health resource groups was excluded from the case-control analyses, as cases will have died in hospital.) The case-control analysis was undertaken on only one year’s data (2005-6) because we assumed that this would be adequate to test the hypothesis and because of the enormous scale of computation required to run a matched analysis on several million records.

### Comparisons with US

We compared the event rates for England in 2005-6 with rates for the US in 2000.^12^ We also compared the results for England for excess length of stay and mortality with US data from the same publication. Although the time periods used for US and England differ somewhat, the paper by Zhan and Miller is the only US paper to have analysed excess length of stay and mortality using a matched case-control analysis; hence we have, for consistency, used it throughout for comparisons with the US data.^12^

## Results

Table 1 presents the numbers of cases, numbers at risk, and rates per 1000 for the nine indicators across three years, involving analyses of some 40 million episodes of inpatient care. As expected, the rates show wide variation between indicators because of differences in the types of events measured. In 2005-6, the rates for the nine indicators ranged from 0.08 (postoperative hip fracture) to 60.34 (obstetric trauma—vaginal delivery with instrumentation) per 1000 discharges. The rates were fairly consistent over time for most indicators, showing little evidence of large or random variation between years. A declining trend was apparent for death in low mortality health resource groups, and an increasing trend for decubitus ulcer and two obstetric trauma indicators: vaginal delivery with and without instrument (these trends were significant at the 1% level). Rates for the remaining indicators were relatively stable.

**Table 1 tbl1:** Indicators of patient safety: rates per 1000 events (95% confidence intervals), England

Indicator	2003-4				2004-5				2005-6		
	No of events	Population at risk*	Rate per 1000		No of events	Population at risk*	Rate per 1000		No of events	Population at risk*	Rate per 1000
Death in low mortality HRGs	3088	5 852 287	0.53 (0.51 to 0.55)		2612	5 916 244	0.44 (0.42-0.46)		2559	6 211 409	0.41 (0.40 to 0.43)
Decubitus ulcer	11 113	1 918 160	5.79 (5.69 to 5.90)		11 860	1 895 455	6.26 (6.14 to 6.37)		13 469	1 878 208	7.17 (7.05 to 7.29)
Iatrogenic pneumothorax	1286	9 677 697	0.13 (0.13 to 0.14)		1244	9 965 794	0.12 (0.12 to 0.13)		1313	10 662 750	0.12 (0.12 to 0.13)
Infections due to medical care	3481	3 696 580	0.94 (0.91 to 0.97)		3649	3 664 065	1.00 (0.96 to 1.03)		3895	3 666 667	1.06 (1.03 to 1.10)
Postoperative hip fracture	292	3 461 567	0.08 (0.07 to 0.09)		267	3 427 111	0.08 (0.07 to 0.09)		291	3 593 089	0.08 (0.07 to 0.09)
Postoperative sepsis	796	350 009	2.27 (2.12 to 2.43)		881	337 804	2.61 (2.44 to 2.78)		885	332 680	2.66 (2.48 to 2.84)
Obstetric trauma:											
Vaginal with instrument	2902	60 664	47.84 (46.1 to 49.58)		3404	63 787	53.37 (51.57 to 55.16)		3966	65 726	60.34 (58.46 to 62.22)
Vaginal without instrument	8493	368 834	23.03 (22.54 to 23.52)		9511	369 708	25.73 (25.21 to 26.24)		11 083	377 070	29.39 (28.85 to 29.94)
Caesarean	338	130 280	2.59 (2.32 to 2.87)		392	132 672	2.95 (2.66 to 3.25)		399	139 304	2.86 (2.58 to 3.15)

HRG= healthcare resource group.

*Cases and patients other than cases, including controls.

Longer lengths of stay and higher mortality in cases compared with matched controls indicate that the measures are discriminatory and indicate the likely occurrence of a safety event. Excess length of stay and mortality is not applicable to the indicator on death in low mortality health resource groups because cases will have died during admission to hospital. The match rate for the remaining indicators was over 75%, except for postoperative sepsis (61%) and postoperative hip fracture (55%) (table 2). For all indicators except one (obstetric trauma—caesarean delivery), cases had significantly longer hospital stays than controls. The excess was greatest for postoperative hip fracture and sepsis (17 and 16 days, respectively) and, as expected, lowest for the obstetric trauma indicators (under one day). Similarly, mortality in cases was significantly higher than in controls for most indicators; the exceptions (again as expected) were the obstetric trauma indicators, where there were no deaths in the matched set for two indicators, and one death for the third indicator. As with length of stay, excess mortality in cases was greatest for postoperative hip fracture and sepsis (18% and 27%, respectively).

**Table 2 tbl2:** Indicators of patient safety: excess length of stay (days) and mortality in cases compared with matched controls, England (2005-6) and US (2000)

Indicator	Match rate %			Excess length of stay (days)						Excess mortality (percent)				
				England			US^12^			England			US^12^	
	England	US		Excess (SE)	P value		Excess (SE)	P value		Excess (SE)	P value		Excess (SE)	P value
Decubitus ulcer	77.2	56.0		9.14 (0.23)	<0.001		3.98 (0.10)	<0.001		13.42 (0.51)	<0.001		7.23 (0.23)	<0.001
Iatrogenic pneumothorax	80.5	66.0		4.35 (0.44)	<0.001		4.38 (0.24)	<0.001		10.59 (1.29)	<0.001		6.99 (0.73)	<0.001
Infections due to medical care	75.3	63.0		11.43 (0.37)	<0.001		9.58 (0.23)	<0.001		5.66 (0.64)	<0.001		4.31 (0.35)	<0.001
Postoperative hip fracture	55.1	51.0		17.09 (1.98)	<0.001		5.24 (0.69)	<0.001		18.20 (3.69)	<0.001		4.52 (1.34)	<0.001
Postoperative sepsis	60.5	33.0		15.90 (0.91)	<0.001		10.89 (0.90)	<0.001		27.07 (2.07)	<0.001		21.92 (1.47)	<0.001
Obstetric trauma:														
Vaginal with instrument	99.6	95.0		0.56 (0.04)	<0.001		0.07 (0.02)	<0.001		—*	NA		0.00	0.32
Vaginal without instrument	99.8	99.0		0.48 (0.02)	<0.001		0.05 (0.01)	<0.001		0.01 (0.01)	0.32		0.00	>0.99
Caesarean	99.2	99.0		0.20 (0.30)	0.49		0.43 (0.14)	0.003		—*	NA		−0.02 (0.02)	0.32

SE=standard error, NA=not applicable.

*No deaths in matched set.

For all indicators, the rates for England were lower than for the US, in most cases by a considerable margin (table 3). The proportional differences were greatest for postoperative hip fracture.

**Table 3 tbl3:** Indicators of patient safety: rates per 1000 events, England (2005-6) and US (2000)

Indicator	England	US^12^
Death in low mortality HRGs	0.41	—
Decubitus ulcer	7.17	21.51
Iatrogenic pneumothorax	0.12	0.67
Infections due to medical care	1.06	1.99
Postoperative hip fracture	0.08	0.77
Postoperative sepsis	2.66	11.25
Obstetric trauma:		
Vaginal with instrument	60.34	224.21
Vaginal without instrument	29.39	86.61
Caesarean	2.86	6.97

HRG=healthcare resource group.

In the Zhan and Miller analysis for the US,^12^ match rates (that is, the proportion of cases with matched controls) were lower than those we obtained for England, except for the obstetric trauma indicators, where both analyses reached near complete match rates (table 2). Match rates are higher in homogeneous situations, such as birth related discharges, than in more complex situations, such as surgery related discharges. Comparisons with the US showed that, for most indicators, the increased length of stay associated with cases was greater in England than in the US, the difference being most marked for postoperative hip fracture. The exception was iatrogenic pneumothorax, where there was reasonable consistency between England and the US. Differences in length of stay between cases and controls for obstetric trauma associated with caesarean delivery were not significant in either case.

Similar patterns were apparent for excess mortality, where levels in England were generally higher than in the US except for the obstetric trauma indicators, which did not show significant patterns because of the low numbers of deaths in both countries. As with length of stay, differences in excess mortality were most marked for postoperative hip fracture.

## Discussion

Routinely collected hospital administrative data are potentially a cost effective source of information on adverse events. Hospital episode statistics cover all NHS inpatient episodes of care in England and are widely used for analyses of clinical outcomes. Some analyses of adverse events coded in routine hospital data for England have been undertaken.^13^ We examined the feasibility of deriving patient safety indicators using hospital episode statistics, assessed whether they are likely to be reliable measures of adverse outcomes by using matched case-control analyses, and compared our findings with those for the US. Our results suggest that the indicators have potential for monitoring patient safety events in the UK but require more in-depth validation of individual cases and better coding of events.

### Limitations of analysis

There are caveats to our findings, some of which are similar to those noted for the US^12^ and are likely to apply also in some other countries.

Firstly, although widely used for analysing quality of care and clinical outcomes,^14^ hospital episode statistics are primarily for administrative purposes, hence the depth of coding can be variable. While coding of procedures and primary diagnoses in the hospital episode statistics is fairly complete, coding of secondary diagnoses (used for several AHRQ indicators) is less complete, hence the adverse outcome rates are likely to be underestimated. In some cases, such as for postoperative sepsis, the number of events is lower than might be expected from clinical experience. This might indicate incomplete coding of events or the existence of alternative systems of recording certain events (for example, dedicated infection control systems) within hospital trusts. A new system of payment (payment by results) has been introduced in England, whereby tariffs are assigned on the basis of treatment and severity. On the basis of experience in other countries, this is likely to improve secondary coding and hence the potential utility of these indicators in the future.^15^ ^16^

Secondly, the translation of ICD-9 diagnoses and procedure codes to ICD-10 diagnoses and OPCS procedure codes could have introduced inconsistencies with the original AHRQ specifications. We have, in consultation with others, refined the translation to capture the key coding requirements, but as thousands of detailed codes for each indicator need cross matching this remains work in progress. Furthermore, international initiatives are underway, including by the Organisation for Economic Cooperation and Development (OECD), to translate the ICD-9 codes for the AHRQ indicators to ICD-10 codes.^11^ We are following these developments and will refine our coding accordingly (although the OPCS procedure codes used in hospital episode statistics are unique to England).

Thirdly, we did not attempt a cross validation of the hospital episode statistics results against patients’ records or other sources of data. Although such comparisons would inevitably be costly, resource intensive, and limited in scale, such validation would be desirable to assess whether the cases identified by the indicators are confirmed patient safety events and will help to refine indicator definitions and support more appropriate use of the indicators on an on-going basis.^17^

Our case-control analysis, based on a national dataset of some 13 million records, provides a pragmatic means of testing the reliability of the indicators and assessing whether they are likely to indicate cases of adverse outcome for patients.

Finally, there are caveats to the comparisons between England and the US, notably because of differences in healthcare systems and patterns of healthcare provision.

### Strengths of analysis

These caveats notwithstanding, our findings are important. Firstly, although different coding systems are used in England and the US, we were able to adapt a subset of the AHRQ indicators for use with hospital episode statistics, demonstrating their technical feasibility.

Secondly, we established that although the indicators might underestimate event rates because of incomplete coding, they have potential as measures of patient safety events. The indicator values were broadly consistent over three years; the lack of random variation indicates some consistency in coding. Excess length of stay in cases compared with matched controls (after adjustment for severity) for all indicators except obstetric trauma—caesarean delivery indicates that cases are likely to have experienced an adverse outcome. Excess mortality in cases compared with controls, observed for all but the obstetric indicators where few deaths occurred, provides further evidence of this.

Our analyses suggest that the indicators are measuring safety related events, and hence support the potential use of datasets such as hospital episode statistics for reporting and monitoring patient safety events. Measures based on administrative data are reported to be generally high in specificity (that is, low rate of false positives) but low in sensitivity (that is, high rate of false negatives),^12^ and our findings support this. Initiatives to show healthcare providers the utility of such indicators for monitoring patient safety, and interventions to improve recording, could therefore increase the value of such routinely collected datasets for patient safety purposes. Until reporting levels improve, however, variations in rates between providers are likely to reflect depth of coding rather than the frequency of patient safety incidents.

Thirdly, our analysis identifies challenges for international comparisons based on these indicators. We found event rates for England were lower than for the US for most indicators. This could be due to various factors but indicates lower levels of recording in England than in the US, where the recording of adverse events or complications is linked to payment systems. The need for improved recording in England suggests that, for the present, increasing rates are welcome because they probably reflect more assiduous attempts to record safety events. Our results were consistent with those for the US in showing longer lengths of stay and higher mortality in cases compared with matched controls for most indicators. That excess lengths of stay were greater in England than the US could be attributable to differences in healthcare systems and the way they are financed. Differences in case mix and clinical practice could also compromise comparability. Furthermore, we did not match for race, as the US analysis did. The longer stays and higher excess mortality in England compared with the US could indicate also that only the most severe clinical events are being recorded in England.

Fourthly, the AHRQ indicators are increasingly being developed internationally. Some OECD countries (Canada, Australia, Spain, Sweden, UK, with some others intending to follow) are piloting these indicators or subsets of them. Outside the US, however, relatively little validation of indicators has taken place. Our work on English data showing differential outcomes between cases and controls will therefore be of international interest. As safety indicators are less well developed in the UK than quality indicators, it also shows that the UK is up to date in testing and applying important emerging initiatives in safety measurement.

Finally, patient safety is a priority for NHS policy makers, commissioners, providers, and regulators. Measuring safety and evaluating the impact of interventions for improving safety, however, poses fundamental problems. Reporting systems designed to enable learning from incidents to be shared across organisations do not capture all incidents and cannot be expected to provide the systematic information needed on rates of occurrence of incidents. Case note reviews are inevitably costly, resource intensive, limited in scale, and don’t allow for benchmarking across providers—a requirement for identifying aberrant patterns. Routine hospital administrative data provide a pragmatic cost effective alternative. Although we have noted some caveats, we have, like others, also shown that such data could potentially be used, alongside other local and national data sources, for improving completeness and quality of coding and monitoring trends in patient safety and local quality improvement initiatives.

There are challenges in developing and using safety indicators, especially in a policy environment promoting publication of performance and quality measures, patients’ choice, competition between providers, etc.^18^ Use of these indicators as “performance measures” could, as with other safety measures, deter coding and reporting and impact negatively on the practical application and utility of these measures. If these risks can be managed by judicious use of the indicators, however, the potential to use routinely collected data for quality improvement will be enhanced for the benefit of patients.

The range of indicators we have described could be extended to include other AHRQ indicators, or potentially other measures, providing a more rounded picture of patient safety. This preliminary work on deriving patient safety indicators for England will also contribute to international initiatives to improve the measurement of safety.^11^

**What is already known on this topic**

Hospital administrative data in the US have been used to derive the AHRQ patient safety indicators
They are used for monitoring national progress in improving the quality of health care, local quality improvement initiatives, and benchmarking of organisations
Several countries are now developing the application of these indicators to support safety monitoring

**What this study adds**

The evidence based AHRQ patient safety indicators can be reproduced for England by using routine hospital administrative data
Case-control analyses, showing longer lengths of stay and higher mortality in cases than matched controls, suggest that the indicators have potential for monitoring patient safety events, as in the US, though levels of event recording need to improve

## Notes

We thank Richard Thomson, Adrian Cook, Jessica Chamberlain, Emma Hawe, and Ann Petruckevitch for their contribution to developing the analyses in the early stages; Frances Murphy for her expertise in translating ICD-9 to ICD-10 and OPCS procedure codes; and the Dr Foster Unit at Imperial College for collaborating on the translation of the indicators, which they did in parallel to us. There have been subsequent modifications to the codes, by them and us, which might account for some differences in rates.

Ethics approval: Not required.

Provenance and peer review: Not commissioned; externally peer reviewed.

## Notes

**Cite this as: ***BMJ* 2008;337:a1702
